# Type 2 Diabetes Patients' Perspectives, Experiences, and Barriers Toward Diabetes-Related Self-Care: A Qualitative Study From Pakistan

**DOI:** 10.3389/fendo.2020.534873

**Published:** 2020-11-27

**Authors:** Allah Bukhsh, Bey-Hing Goh, Edward Zimbudzi, Clement Lo, Sophia Zoungas, Kok-Gan Chan, Tahir Mehmood Khan

**Affiliations:** ^1^School of Pharmacy, Monash University, Subang Jaya, Malaysia; ^2^Institute of Pharmaceutical Sciences, University of Veterinary and Animal Sciences, Lahore, Pakistan; ^3^College of Pharmaceutical Sciences, Zhejiang University, Hangzhou, China; ^4^Biofunctional Molecule Exploratory Research Group, School of Pharmacy, Monash University Malaysia, Bandar Sunway, Malaysia; ^5^Malaysia School of Public Health and Preventive Medicine, Monash University, Melbourne, VIC, Australia; ^6^School of Public Health and Preventive Medicine, Monash University, Melbourne, VIC, Australia; ^7^Department of Nephrology, Monash Health, Melbourne, VIC, Australia; ^8^Monash Diabetes, Monash Health, Melbourne, VIC, Australia; ^9^Division of Genetics and Molecular Biology, Faculty of Science, Institute of Biological Sciences, University of Malaya, Kuala Lumpur, Malaysia; ^10^Guangdong Provincial Key Laboratory of Marine Biology, Institute of Marine Sciences, Shantou University, Shantou, China

**Keywords:** type 2 dabetes, self-care, self-monitoring blood glucose (SMBG), barriers and facilitative factors, exercice, diet

## Abstract

**Objective:** This study aimed to qualitatively explore perspectives, practices, and barriers to self-care practices (eating habits, physical activity, self-monitoring of blood glucose, and medicine intake behavior) in urban Pakistani adults with type 2 diabetes mellitus (T2DM).

**Methods:** Pakistani adults with T2DM were recruited from the outpatient departments of two hospitals in Lahore. Semistructured interviews were conducted and audiorecorded until thematic saturation was reached. Two researchers thematically analyzed the data independently using NVivo® software with differences resolved by a third researcher.

**Results:** Thirty-two Pakistani adults (aged 35–75 years, 62% female) participated in the study. Six themes were identified from qualitative analysis: role of family and friends, role of doctors and healthcare, patients' understanding about diabetes, complication of diabetes and other comorbidities, burden of self care, and life circumstances. A variable experience was observed with education and healthcare. Counseling by healthcare providers, family support, and fear of diabetes-associated complications are the key enablers that encourage study participants to adhere to diabetes-related self-care practices. Major barriers to self care are financial constraints, physical limitations, extreme weather conditions, social gatherings, loving food, forgetfulness, needle phobia, and a hectic job.

**Conclusion:** Respondents identified many barriers to diabetes self care, particularly related to life situations and diabetes knowledge. Family support and education by healthcare providers were key influencers to self-care practices among Pakistani people with diabetes.

## Introduction

Diabetes mellitus (DM) is one of the most challenging health care issues of the twenty first century. Type 2 diabetes (T2DM) is the most common form of diabetes and affects more than 90% of people with diabetes. In addition to genetic pre-disposition, physical inactivity,obesity, and unhealthy eating habits are significant risk factors for T2DM (1, 2). In Pakistan, the diabetes prevalence rate is currently 6.9%, but it is projected to reach 15% by 2040, giving Pakistan the fourth highest prevalence of diabetes globally (3).

Self-care practices have been positively correlated with good glycemic control and significant reductions in the progression and development of complications associated with diabetes (4, 5). Diabetes-related self-care practices include healthy eating, being physically active, self-monitoring of blood glucose, and regularly taking prescribed medications (6).

Adherence to recommended diabetes self-care activities is important in achieving the desired glycemic control and reducing diabetes-related complications (7, 8). Despite known clinical benefits associated with diabetes self-care activities, a number of studies report poor adherence to recommended diabetes-related self-care practices (9–11). Adherence to self care depends on patients' lifestyle behaviors, such as adopting healthy eating practices and physical activity (12). Inadequate disease knowledge; poor communication with healthcare providers; and psychological factors, such as depression, are frequently reported barriers to recommended self care (7). Self-care education, family support, and problem-solving skills are commonly suggested facilitators for improving diabetes self-care practices in people with diabetes (1, 13, 14).

To date, several quantitative studies have examined patient knowledge levels and self-care practices among people with diabetes in Pakistan (9, 15–18). Psychological and cultural factors are frequently reported barriers to diabetes self-care in Pakistan (15, 18). These studies reveal gaps in knowledge regarding diabetes and highlight the importance and feasibility of self-care educational interventions (9, 19). Evidence synthesized from a recent network meta-analysis shows that self-care educational interventions are effective in achieving desired clinical outcomes of people with diabetes; for example, a significant reduction in glycated hemoglobin (HbA1c) levels, systolic blood pressure, and lipid profile was observed (20).

Although there are a few published qualitative studies that address self-care experiences (21, 22) and cultural perceptions (23) of people with diabetes from rural areas of Pakistan, none has explored the perspectives and experiences of self care in adult Pakistani people with T2DM residing in urban areas of Pakistan. Second, diabetes has become a serious health challenge for low- and middle-income countries, such as Pakistan, where self-care aspects of diabetes are not properly discussed with patients (21). Exploring T2DM patients' perspective in depth and identifying facilitators and barriers to diabetes self care will not only yield new knowledge regarding self care among this population, but will also help to prioritize treatment targets and design strategies, such as tailored self-care educational interventions, specific to the culture and needs of Pakistani people with diabetes. The objective of this qualitative study is to provide insights into the experiences, behaviors, and barriers to self-care practice among urban Pakistani adults with T2DM.

## Methodology

### Study Design and Setting

This study utilizes qualitative research methods to comprehensively explore patients' perceptions and behaviors toward disease-management practices. In-depth interviews were conducted, using a flexible, semistructured guide with an open-ended questioning approach (24), among people suffering from T2DM at Akhuwat Diabetes Clinic Lahore and Awan Medical Complex Lahore, Pakistan (2016–2017).

### Ethical Approval

This study was approved by the Monash University Human Research Ethics Committee (MUHREC, Approval Number 7767) and the data collection centers in Pakistan. Informed written consent was obtained from all study participants after providing them with a verbal and written explanation about the purpose and required procedures of the study. Participation in the study was voluntary, and participants were told that they could withdraw from the study at any time. Only verbal consent could be obtained from illiterate participants. Confidentiality was maintained by using study codes. Data access was restricted to study researchers only. The ethics procedures of the study comply with the Declaration of Helsinki.

### Study Sample and Data Collection

Patients with T2DM were recruited using convenience sampling. Inclusion criteria were (1) Pakistani national of age more than 30 years, suffering from T2DM for more than 1 year (to allow patients with T2DM to familiarize with facilitators and barriers toward their diabetes self care) and (2) willingness to be interviewed in Urdu language (audiorecorded) within the hospital premises. Patients who were diagnosed with other diabetes types, pregnancy, and cognitive impairments such as dementia were excluded from the study. In order to identify patients who met the inclusion criteria, the first author screened the patients at both diabetes centers by reviewing their medical record files and after consulting physicians who were attending these patients. Patients meeting the study's inclusion criteria were physically approached by the first author while they were waiting for their appointments with the physician at the diabetes care centers. Sixty-four patients met the inclusion criteria. After explaining the purpose and process of the study to the eligible patients, consent was obtained for an audiorecorded interview at the clinic. Thirty-seven eligible patients (response rate of 57.8%) agreed to participate.

Interviews were conducted until thematic saturation was reached (25). The interviews were conducted by a single interviewer (a researcher who was not involved in the provision of healthcare to the participants in the past) to minimize interindividual variability.

### Contents of the Interview

A semistructured interview guide (attached in the Appendix) was developed after a literature review (1, 4, 7, 8, 26–31) and discussion with academic and clinical diabetes experts to ensure that key areas of diabetes self care were covered in a culturally acceptable manner. Key domains of self care on which data were collected included knowledge and practices toward diabetes medicine, self-monitoring of blood glucose, healthy eating, physical activity, and continuity of care. It was then piloted (sample size *n* = 2) to ensure that the content of the interview guide sufficiently covered all domains of diabetes self care. Pre-test interviews were not included in the final analysis. Widely framed and open-ended questions gave ample opportunities to the study participants to share their personal experiences and factors that facilitate and impede their practices toward diabetes self care. Participants were also encouraged to shape their own narratives and share anything further relevant to the topic. In addition, during the interview, relevant keynotes were also taken so as to document key observations and issues.

### Analysis

All interviews were audiotaped in the Urdu language before being translated and transcribed verbatim to English language by the first author (AB). The transcribed interviews were reviewed by a second researcher (TMK) to ensure transcriptions were accurate, complete, and unbiased (32–34).

After familiarizing themselves with the data, two researchers (AB, EZ) independently coded the data by using NVivo® software (version 11 plus). A generic thematic analysis approach (35) was used to categorize the codes through several iterations. Themes were identified from the coded data. Discrepancies in coding between the two investigators were resolved through consensus with a third author as required (CL). Emergent themes were then discussed among all the authors for consistency and to minimize the bias.

## Results

Thirty-two patients with T2DM participated in the study, of which 21 (65.5%) were female. The age of participants ranged from 35 to 75 years, and all spoke Urdu as their first language. Ten respondents reported being employed, and most of the female respondents were housewives. Table 1 lists the demographic details of the study participants (demographic details of individual participants are provided in the Appendix).

**Table 1:** Demographic characteristics of the participants (*N* = 32).

**Characteristic**	**Mean (*SD*) or percentage (*n*)**
Age (years)	54.81 ± 9.59
Gender	
Male	34.5% (11)
Female	65.5% (21)
Working status	
Housewives/stay at home	53.1% (17)
Business	6.2% (2)
Doing job	31.2% (10)
Retired	9.5% (3)
Education (years)	
No formal education	34.4% (11)
Primary level	21.9% (7)
Secondary level	21.9% (7)
High secondary level	9.3% (3)
University level	12.5% (4)
Duration of diabetes (years)	9.7 ± 7.58

*SD, Standard deviation*.

**Figure 1:**
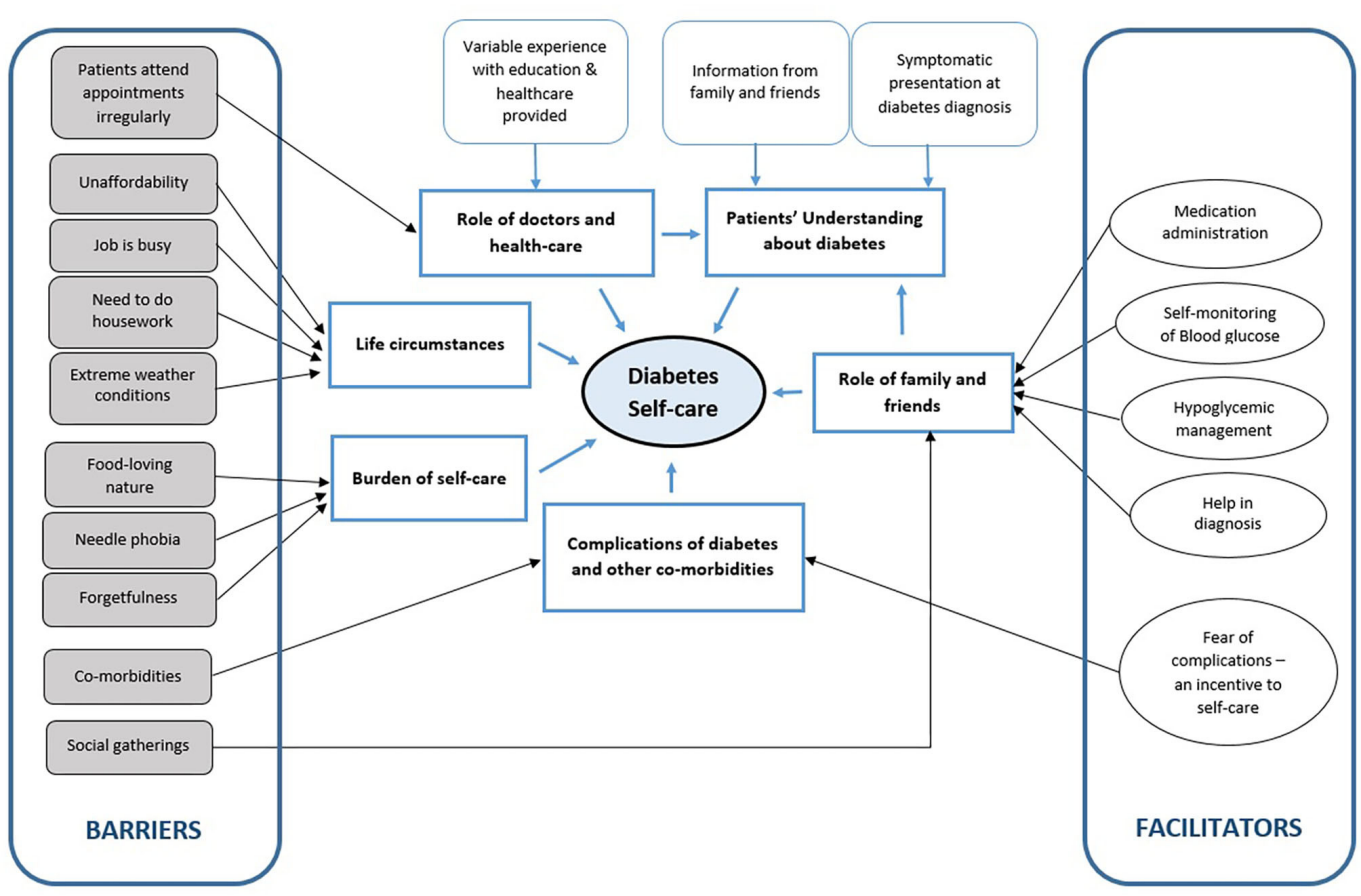
Thematic presentation of factors influencing self care in diabetes.

Six themes were identified after an in-depth analysis of the participants' interviews. The list of themes and subthemes are presented in Figure 1.

## Theme 1: Role of Family and Friends

### Medicine Administration

Getting support from family members is one of the important determinants of compliance with medication-taking behavior. Family support in the form of reminders to take medications and help in medicine identification and administration are important facilitators to diabetes self care. Some participants shared that they can identify their medicines only by color or shape, whereas their family members (e.g., spouse and children) helped in identifying and administering their medicines.

“*My son has the responsibility to give me medicines. I can't recognize and remember my medicines, as every time doctors change my medicines. My son buys medicines for me and has the responsibility to administer me.” (P12; Male)*

Another male participant (P4) further added

“*I started with Glucophage (Metformin), and now on insulin. I can identify my medicine from its color, but mostly my wife and my child administer me medicine. My children help me in identifying my medicine.”*

### Self-Monitoring of Blood Glucose

Participants repeatedly discussed how family members support and motivate them to practice regular blood glucose testing. Family support in the form of glucometer handling is a key enabler reported by most of the respondents for self-monitoring of blood glucose (SMBG). One participant stated

“*I check [blood glucose levels] at home twice a week, I have glucometer at home, I cannot operate it, but my daughter-in-law does it for me.” (P1; Female)*

Respondents' children were frequently mentioned family supports in diabetes self care. Many participants stated that their children helped them in performing blood glucose checks.

“*I have glucometer at home, but I cannot operate it, my children often do it for me.” (P15; Male)*

Another male participant (P4) further added this:

“*I don't feel any problem as my wife can use a glucometer, she is educated and can operate it.”*

### Hypoglycemia Management

Some participants mentioned family members as a source of information for their hypoglycemia:

“*When you have so many diabetic patients in your family, you will become a doctor because of their disease experience. No one told me about hypoglycemia, I learned from my family members about the symptoms of hypoglycemia and keep a sugar candy or sugar sachet with me always.” (P18; Female)*

Very few participants discussed the assistance provided by family members during their hypoglycemic episodes.

“*Once I felt such condition [hypoglycemia], when I was sleeping, felt like shaky, tired and weak, I called my daughter-in-law, she brought for me lemonade with plenty of sugar in it. My son got frightened of my condition, but after some time I became normal, before they took me to hospital.” (P1; Female)*

### Help in Diagnosis

Information and support from the family is an important source, not only for diabetes self care but in diagnosing interviewees' diabetes as well. A few participants reported that they came to know about their diabetes symptoms because of their family members and friends who were already suffering from diabetes.

“*Many of my family members and relatives are suffering from diabetes and that's why I knew about the symptoms of diabetes.” (P3; Female)*

Another female participant (P9) described the importance of counseling from her family member:

“*One of my family members who also had diabetes counseled me that I should take proper diet, follow doctor advice and regularly take my medicine, only then my diabetes will remain under control.”*

Another female participant (P32) discussed how her neighbor counseled her to get checked for blood glucose levels.

“*Thirteen years before I suffered from weakness and body pains, upon discussion with my neighbor, who was already suffering from diabetes, advised me to get checked from a physician for diabetes. Upon blood sugar check-up form a private hospital at Lahore, it was revealed that my random blood sugar was 320 mg/dL.”*

### Social Gathering

Diet management is one of the most problematic self-care behaviors as described by the majority of participants. Among the several challenges for adopting a healthy lifestyle, the most commonly mentioned was difficulty in maintaining a healthy diet. Participants specifically mentioned that it was difficult to follow diabetes-related dietary recommendations when food was presented in wedding ceremonies and social get-togethers.

“*On marriage ceremonies and on other social gatherings it is difficult for me to manage my diet, due to plenty of sugar-rich food served there.” (P15; Male)*

Some participants highlighted the unavailability of diabetes-specific food in social gatherings, especially marriage ceremonies.

“*In marriage ceremonies, no food is available for diabetes patients, so I tend to eat whatever served.” (P22; Female)*

Problems with adhering to their medicine intake in social gatherings were also reported by the study participants.

“*At maximum, I skip one dose, only when I am out of my house to attend some family get-together, but I take the rest of the medicines properly on my return to home. It's very rare that I forget my dose.” (P13; Female)*

A male respondent (P26) further added in to this:

“*Whenever I go out to attend family parties, I forget to take my medicine.”*

## Theme 2: Role of Doctors and Healthcare

### Variable Experience With Education and Healthcare Provided

Education from physicians is one of the most important determinants for change in self-care practices. Counseling and education from physicians not only improved the knowledge of the study participants, it was a source of encouragement for them as well. Many participants acknowledged and clearly valued their physician's counseling regarding medication adherence and adopting a healthy diet plan.

The majority of the study participants mentioned that they were strictly adhering to their prescribed antidiabetic medications' schedule as compared to other self-care practices.

“*I take my medicines regularly because my doctor advised that whether I take meals or not, I must take my medicines regularly.” (P2; Female)*

A female participant (P24) mentioned

“*Doctor wrote for me clearly and in bold on medicine pack and blisters, and my children can read that easily.”*

Participants indicated that their healthcare providers were a major source of encouragement for medication adherence.

“*Initially, I use to take my antidiabetic medicines irregularly because whenever I got my blood sugar levels tested, it used to be normal (140-150), so I did not pay heed toward my regular medicine intake. But my doctor advised me to take it regularly otherwise my disease will worsen. Now I take my medicine regularly.” (P3; Female)*

The majority of patients reported being properly counseled by their physicians to do regular exercise and about its importance in their diabetes self care.

“*Doctor guided me to do daily 30 minutes' walk early morning. It's the only exercise I have to do, and I do it regularly.” (P30; Male)*

Another male participant (P15) further added

“*I do 45 minutes' walk daily. My doctor advised me, and I am aware of its importance as well.”*

Participants were aware of the need for dietary modification for their diabetes self care. Most of them commonly described being counseled either verbally or in writing by their healthcare providers about dietary modification during their visits for medical checkups.

“*Doctor gave me a diet plan, which is hanging on our kitchen wall, I follow it mostly. I avoid sugar, sweets, soft drinks and meat.” (P10; Female)*

Another female participant (P13) mentioned

“*My doctor gave me a diet plan and I follow it with true spirit. I know about my dietary precautions and I have been counseled by my doctor properly. I avoid sugar, rice, carbonated sugar drinks and beef, but take mutton sometimes.”*

Participants elaborated that they had received information on “what to eat and how to eat,” and they had been advised “to eat more green leafy vegetables and locally available fruits.” Interviewees stressed the fact that they should follow what is being advised to them for leading a healthy life and appreciated that a change in dietary behavior is good for their health.

“*Doctor advised me to avoid rice, seafood, potatoes. Eat less but more frequently.” (P22; Female)*

Participants were aware and educated about the importance of a portion diet and ideal food choices for diabetes. A female participant (P21) shared her views:

“*I am aware of my diet planning, as the doctor guided me well about it. I know the kind of food which is beneficial and harmful to me.”*

Some participants mentioned that they gained knowledge about hypoglycemia, associated symptoms, and its management from their physicians:

“*Doctor told me that if I feel restless and sweating, I should take something sweet and always keep a sugar candy with me.” (P16; Female, P14; Female; P30; Male)*

Another male participant (P20) added

“*My doctor counseled me about the possibility of being hypoglycemic, as I was prescribed with two types of capsules and insulin for my diabetes.”*

Those who received education about hypoglycemia were aware of its emergency management at home:

“*Now I am aware that I will eat something sweet whenever my blood sugar levels are low.” (P29; Female)*

Very few participants were aware of keeping sugar with them for emergency management of hypoglycemia:

“*Quite often I experience fatigue, hunger, palpitation. I know such condition is because of low blood sugar levels and I eat dates or anything sweet available at home.” (P32; Female)*

At the same time, a different experience with healthcare providers was shared by some of the participants. According to these interviewees, the self-care aspect of diabetes management and the significance of routine checkups were not discussed with them by their physicians during their consultations.

“*Doctors did not advise me to get my eyes and kidneys checked regularly.” (P3; Female)*

Another female participant (P5) added

“*I did not go for regular checkup of my kidney and eyes, as my doctor did not advise me for these check-ups.”*

One female participant (P31) mentioned

“*I get my eyes and kidneys checked myself, but doctors never asked me to do so. Doctors use to ask me about my previous medicine intake behavior but never asked me about my dietary habits.”*

A few participants expressed their feelings that they were not treated well by their physicians.

“*Doctors never guide me about my medicines, if I ask them, they simply reply, ask dispensers for this.” (P2; Female)*

A female participant (P24) shared her experience:

“*Doctors don't give me sufficient time, just write a prescription for me…”*

Another female participant (P25) shared her expectations toward her physician:

“*I want my doctor to listen to my medical concerns in detail and counsel me properly.”*

A small number of interviewees also reported that their physicians never asked them about their routine diet and medicine intake behaviors. They insisted on getting more information from their physicians about diet planning and lifestyle modifications.

“*Doctor never asked me about my diabetes medicines intake and dietary precautions.” (P26; Male)*

Participants showed their interest in receiving detailed information from their physicians about their lifestyle modifications:

“*Doctor never asked me about my diet and medicine routine. I want my doctor to guide me about my diet plan.” (P22; Female)*

Another male participant (P30) further added this:

“*Doctors never asked me about my diet and medicine intake. Doctors write in English language which I cannot understand, so I request him to write it for me in Urdu language.” (P30; Male)*

One male participant (P20) shared his experience of not receiving written diabetes educational material from the healthcare facility:

“*I did not receive any written material from clinics regarding diabetes.” P20*

Despite the serious nature of hypoglycemia, most of the participants indicated that they did not receive education about hypoglycemia and its management from their physicians.

“*My doctor never told me about the symptoms and management of hypoglycemia.” (P5; Female)*

Some participants mentioned that, although they were not properly educated about hypoglycemia and its management, they knew to eat something sweet in case of hypoglycemia:

“*I was not told about this (hypoglycemia). But whenever I feel that I am having a low blood sugar level, I take sugar at my own.” (P1; Female)*

Another male participant (P12) further added

“*I don't know the symptoms of a low blood sugar levels and did not experience it (hypoglycemia) yet. But I know I will eat sugar candy.”*

### Patients Attend Appointments Irregularly

Most of the study participants shared that they were more likely to consult their physicians when they experienced ill symptoms or in case of disease severity.

“*I visit my doctor only in case I feel I am having low blood [blood pressure], but most of the time blood [blood pressure] is normal but my sugar level is high.” (P8; Female)*

Another female participant (P29) shared her experience:

“*I consult doctor irregularly, only when I am facing some medical problem.”*

Some patients shared that they visited their physician only in case of medical emergency:

“*Without any medical issue, I never visit my doctor. Today I am visiting my doctor after about 6 months. As I believe if I am taking my medicines regularly and without any emergency condition, there is no need to visit my doctor.” (P19; Female)*

One female participant (P28) reported

“*I visit my doctor only in case of a medical emergency and when my sugar levels are not being controlled.” P28*

A considerable number of study participants mentioned that they visit healthcare facilities only to get their medicines refilled free of cost rather than for a routine medical checkup.

“*I visit a hospital after one month, just to take my medicines for diabetes and return home, no one (from hospital) inquires me about my disease and how I am taking my medicines and diet.” (P2; Female)*

The same thoughts were shared by other participants:

“*I visit my doctor to refill my prescription.” (P9; Female)*

“*I visit my doctor every month because on the visit I get free (medicine) insulin.” (P15; Male, P22; Female)*

A female participant (P1) shared her experience:

“*I visit my doctor after every 15 to 30 days, as I have to get insulin free of cost from the diabetic clinic after consultation. Only once I got checked for my kidney and eyes.”*

Only a few of the study participants mentioned that they visited their physicians for routine checkups.

“*I visit my doctor after every three months for a routine checkup or in case I have some problems. The doctor advised me to get my eyes checked after every 3 months because my vision is being adversely affected by my diabetes.” (P11; Male)*

One female participant (P24) mentioned that her physician educated her about the importance of regular medical checkups:

“*I visit the doctor after every month, irrespective of having any medical issue. Doctors advised me to get myself checked regularly for follow-up purposes.”*

Blood glucose testing was also a reported reason for a healthcare facility visit by a few study participants.

“*After every week, as I have to get my blood sugar levels checked. I visit my doctor for my lab reports review too.” P7*

## Theme 3: Patients' Understanding About Diabetes

### Information From Family and Friends

When the participants were asked how they were diagnosed with diabetes, many of them mentioned their family members and friends as primary sources of information for their diabetes.

“*People shared with me that excessive urination was due to the weakness of bladder, some said I should drink plenty of water, and some advised to take soft-drinks, but no one told me about diabetes.” (P1; Female)*

Some participants perceived diabetes as a curable disease.

“*My husband told me that I will be cured of diabetes, and I was not upset, as I did not know about it, and was expecting that it will be cured.” (P13; Female)*

A similar experience was shared by another male participant (P6).

“*I realized that things will be normal, and nothing is going to happen to me. I was expecting that my diabetes will cure after taking medicine for a couple of months. But now after one year, I have realized that I have to take medication and diet control for rest of my life.”*

A few myths were shared by the study participants regarding the cause of their diabetes.

“*I thought I became diabetic patient due to herbal medicines, which I was taking for treatment of my kidney stones treatment.” (P23; Female)*

According to a female participant (P5), glucose infusions were the cause of her diabetes.

“*Due to excessive administration of glucose infusions, when I got gallbladder surgery around 10 years back, was the reason for my diabetes.”*

On the other hand, a female (P9) participant thought that depression was the cause of her diabetes

“*I became diabetic patient due to depression of my friends' death, the initial symptoms I got were excessive urination.”*

### Symptomatic Presentation at Diabetes Diagnosis

The majority of participants described experiencing a variety of ill symptoms, such as frequent urination, fatigue, and body pains, especially in their lower limbs (which they did not attribute to diabetes), which drove them to visit a doctor. For instance, according to male participant P1,

“*Due to pain in my legs and frequent urination, I consulted the doctor and was diagnosed with diabetes.”*

Some patients went for other disease checkups and were diagnosed with diabetes:

“*Due to vertigo and fatigue I visited my family physician along with my husband, I was diagnosed with diabetes, at that time I did not even know what diabetes is?” (P13; Female)*

Another female participant (P17) shared her experience of diabetes diagnosis:

“*I was admitted into a hospital due to dengue fever, and was diagnosed with diabetes, at that time my blood sugar was 300 [mg/dL].”*

Delayed wound healing also led to a diabetes diagnosis in a few participants:

“*I had a wound on my leg which was not healing despite taking antibiotics. Upon complete lab testing, I was diagnosed with diabetes, which was the reason, why my wound was not healing.” (P21; Female)*

## Theme 4: Complications OF Diabetes and Other Comorbidities

### Fear of Complications—An Incentive to Self Care

Illness perception is a key factor that seems to influence participants' decisions to adhere to their recommended medications and SMBG levels. Irrespective of diet and lifestyle adherence, most of the study participants mentioned that they were strictly adhering to their prescribed antidiabetic medication schedule, which they attributed to fear of ill symptoms and complications associated with poor diabetes control. The appearance of body pains, fatigue, and troublesome frequent urination were the most frequently mentioned fears by the respondents.

“*If I discontinue my medicine my blood sugar level will increase and I will suffer from body pains again, that is why I never think of discontinuing my therapy.” (P16; Female)*

Fear of acquiring diabetes-related complications is the most notable patient concern if they do not adhere to their recommended medicines.

According to a female participant (P17),

“*I know my diabetes get worse if I did not take care of it.”*

A female participant (P2) further added to this:

“*As I take my medicines regularly, I can walk and perform my daily life activities, otherwise, it becomes difficult for me to walk even.”*

Staying healthy and keeping blood sugar levels within normal limits are key enablers to perform blood sugar testing as reported by many respondents.

“*I check my blood glucose levels every 2 to 3 days, especially when I am not feeling well.” (P4; Male)*

### Comorbidities

Comorbidities can limit one's ability to self care. Some participants have a plethora of comorbidities, ranging from joint problems to body pain and fatigue, which restricts them from pursuing regular physical activity. Body pains, especially in the legs and feet, are the most commonly reported barrier to maintaining daily exercise. Some remarks by patients that brought forth this point are

“*I have been doing a walk regularly. But for the last 6 months, I am not doing a regular walk, because of pain in my legs.” (P11; Male)*

Another female participant (P18) further added to this:

“*For a long time, I am not doing exercise because of the pain in my legs and feet. I got weak now and cannot exercise due to muscles pain.”*

Many of the study participants mentioned that they tried to do exercise at the same intensity and duration as their healthcare providers advised them, but fatigue, body pains, and injuries to the knee and hip bone hamper their ability to sustain a regular exercise.

“*My doctor advised me to do walk, but I can't walk or do any sort of exercise because of my hip bone problem.” (P21; Female)*

Some of the participants reported that they were no longer able to do exercise because of their comorbid condition, such as heart problems.

“*Before having a heart attack I used to do exercise. Now after having a heart attack I cannot do exercise. I feel like my muscles become weak and get exhausted with a light walk.” (P4; Male)*

## Theme 5: Burden OF Self-Care

### Loving Food

Cravings for particular types of foods, such as desserts, make it difficult for several respondents to avoid sugar and other foods that are restricted in diabetes. One female participant (P1) shared her experience:

“*I don't strictly avoid sugary food, sometimes I do eat those. I love to eat sweets. One month back someone gifted a pack of sweets to us, my family members kept it inside the refrigerator, so that I should not be aware of it, but once I looked at it, I ate it in the absence of my family and nothing happened to me.” (ha-ha. patient laughs aloud)*

Participants voiced frustration with sacrificing their food liberty. In regard to dietary issues as barriers to their diabetes self care, one participant stated

“*I feel it very difficult to sacrifice my food liberty. I am a food lover and it is hard for me to live a tasteless life, so quite often I enjoy the food of my choice.” (P20; Male)*

A female participant (P32) shared her experience:

“*Sometimes I do take sweets, for example, I am fond of a traditional sweet ‘Halwa,' and I eat it despite knowing the fact that it is full of sugar.”*

### Needle Phobia

Fear of pain associated with fingertip pricking is a reported barrier for frequent SMBG by a few participants. One participant stated

“*Doctors advised me to check (blood glucose levels) daily, but I am afraid of needle prick.” (P24; Female)*

Another male (P30) mentioned a fear of the needle linked with his insulin injections:

“*Because of my extensive insulin therapy, I am afraid of needle now,”*

### Forgetfulness

A few respondents claimed to be negligent in taking their medicine and refilling prescriptions.

“*I forget to take medicines sometimes and don't take when medicine stock is finished at home.” (P14; Female)*

A male participant (P15) further added

“*I take my medicines regularly, but sometimes I forget to take, as I feel I am losing memory due to diabetes. I do take insulin with me when I travel outstation and keep it in the refrigerator but still, sometimes I tend to forget.”*

## Theme 6: Life Circumstances

### Unaffordability

Affordability of healthy food is a commonly mentioned barrier to adopting a recommended diet plan.

“*There is no question about the diet plan and food restrictions for a person who can hardly afford two times meal for her, I can hardly manage to buy 250 ml of milk and four slices of bread a day, which I utilize for my whole day and sometimes my neighbors give me bread for my lunch.” (P31; Female)*

A female participant (P9) further added

“*I can't follow the diet plan because the food mentioned in it is difficult to understand and afford. Only rich people can afford such menu and time schedule, like two servants are required to serve it and make you stick with that diet plan. I follow some parts of the diet plan, which I can understand and afford.”*

A lot of the interviewees admitted that, despite receiving a diet plan, they ate whatever was cooked at their home:

“*My doctor gave me a diet plan. I can understand it but cannot follow it due to unaffordability issues, so eat the food which is cooked at home.” (P16; Female)*

The cost associated with blood glucose monitoring is also reported as one of the reasons why participants did not practice blood glucose testing regularly.

“*I don't have a blood sugar checking facility in my village … above all it is expensive too, and hard for me to afford.” (P18; Female)*

Even so, the cost associated with insulin is also reported as a reason for medicine non-adherence:

“*Insulin is very effective, during my job I was provided with free insulin, but as of now I am retired from my job, it's expensive and difficult for me to afford. But the person who discovered insulin God bless him, as he saved millions of lives like mine.” (P15; Male)*

A female participant (P18) further added

“*I don't have a blood sugar checking facility at my village and its available far away from my home and above all it is expensive and difficult to afford.”*

### Job Is Busy

Hectic work schedules and other job-related responsibilities and obligations are reasons why some of the participants could not adhere to their recommended healthy eating habits. According to one respondent,

“*I know to eat less and more frequently, but due to the nature of my job, I cannot adopt it.” (P26; Male)*

In regard to the medicine intake schedule, one male participant (P12) mentioned

“*I do take medicines regularly but due to business when I have to go out of the city, I don't take medicine stock with me for days”*

Participants indicated that it was difficult to incorporate recommended physical activities into their daily life due to the hectic nature of their job:

“*I do not do regular exercise due to my overburdened job nature.” (P26; Male)*

Some participants further added to this, citing fatigue due to the tiring nature of their job, which impeded their exercise routine:

“*I have no spare time from my work to do exercise, after the job I am so tired to even think of exercise” (P8; Male)*

### Need to Do Housework

Several participants expressed that they are not required to do regular exercise as they think that their routine life activities are a fair substitute for their exercise. One response pointing this aspect is

“*I perform my household work which I consider enough replacement for my exercise.” (P21; Female)*

Another female participant (P9) further added to this:

“*My working at my home and kitchen is my exercise, as I am the only working lady at my home.”*

### Extreme Weather Conditions

Some respondents mentioned that the harsh weather conditions during winter and summer impede their engagement in regular physical activity.

“*I regularly go for a morning walk for about 25 to 30 minutes, except when I had severe body pains and during unfavorable weather conditions, like rain, hot and cold weather. Nearly one month during winter I do not go for a walk due to foggy weather.” (P32; Female)*

A similar experience was shared by male respondent (P20):

“*I walk for around one hour daily. But for the last few weeks due to extreme weather conditions [hot weather], I am avoiding my routine walk.”*

## Discussion

This study explores the perceptions, experiences, enablers, and barriers to diabetes self care by patients with T2DM living in urban areas of Pakistan. Diabetes self care requires adopting a healthy lifestyle in addition to adhering to prescribed medicine and regular blood glucose testing. Overall, participants exhibited a poor knowledge about diabetes, complications associated with diabetes, and the importance of healthy diet and regular exercise.

Counseling by healthcare providers and family support assists the participants for better disease management. Those who are unsuccessful in adopting self care identified several barriers, especially adhering to a healthy diet plan and physical activity. Previously published studies focus on views and self-care experiences of people with diabetes living in rural areas of Pakistan (21, 22).

Support from family members promotes self-care practices among study participants in a variety of ways, including medicine identification, medication administration, blood glucose testing, and managing hypoglycemia. Several participants remarked that they had difficulty in medicine identification and glucometer handling for their blood glucose testing although assistance and encouragement provided by their family members facilitated them in medication adherence. The importance of family support as an enabler to improve medication adherence and blood glucose testing in people with diabetes living in rural areas is reported in both low- and middle-income (4, 36) and high-income countries (1, 8).

Participation in social gatherings, such as wedding ceremonies, is a frequently shared barrier to self care by the participants, because the food served at such occasions is highly unsuitable for people with diabetes. Our results are consistent with those of Tewahido and Berhane (37) and Lekoubou et al. (38), which indicate food related to sociocultural norms poses a significant barrier to effective diabetes management. Healthy eating practices can be improved in people with diabetes by considering the cultural aspects of food and individuals' taste preferences (39).

Living as a joint family is a part of Pakistani culture, and the eating behaviors of family members can influence the eating habits of diabetes patients in the family (40). Cultural norms coupled with affordability issues are posing a lot of difficulties for Pakistani people with diabetes in adopting healthy eating practices (21) as is evident from the fact that most of the study participants mentioned that they had to eat whatever was cooked at home. Our results are also consistent with those of Ansari et al. (21), who find a lack of social and family support in dietary adherence of middle-aged diabetic patients residing in rural areas of Pakistan.

Variable experience with healthcare providers and disease education is shared by the participants. Most of the participants describe education by healthcare providers as one of the major facilitators to their diabetes self care. Interviewees expressed that their physicians were not only a source of information for their medicines, blood glucose level monitoring, diet planning, and hypoglycemia management, their encouragement also supported the participants in improving medication compliance, physical activity, and healthy eating habits. These findings are consistent with many published studies in which the knowledge and reassurance provided by healthcare providers assists participants in behavior modifications and managing their diabetes in a better way (8, 41).

At the same time, a different experience was shared by other interviewees. Concerning the patient–doctor relationship, some participants shared the fact that they were not educated about various aspects of self care. A similar experience of dissatisfaction with physicians' attitudes has been reported by Ansari et al. (21), in which the self-care component of disease management was not discussed with Pakistani people with diabetes dwelling in rural areas.

Hypoglycemia is an acute medical complication and requires immediate identification and management to minimize vital organ damage. Although the incidence rate of hypoglycemic episodes is very low in T2DM patients in the first few years of their diagnosis, it can increase up to 25% with disease progression and the patient's shift to insulin (42). While attaining the target of glycemic control in people with diabetes, prevention of hypoglycemia remains one of the main hurdles (43). In our study, only a few participants shared that they were instructed by their physicians about hypoglycemia and its management at home. Whereas the majority were unaware of the symptoms of hypoglycemia and its management and urged their physicians to guide them about hypoglycemia and its emergency management at home. Educating people with diabetes about symptoms of hypoglycemia, associated risk factors, and preventive strategies will result in achieving desired health-related outcomes (44).

Delayed and irregular access to healthcare services leads to poor disease management and increased morbidity (45, 46). In our study, an irregular pattern of healthcare access was reported by the participants; patients visit healthcare facilities only when they are experiencing ill symptoms, in case of a medical emergency, to refill their prescription for free medicine, and for free blood sugar testing. Unaffordability, low education levels, and poor counseling by healthcare professionals about the importance of regular medical visits are the main barriers to regular healthcare facility visits.

Surprisingly, the majority of interviewees stated that their diabetes diagnosis was unexpected: They were suffering from diabetes symptoms, but they did not know that these symptoms were due to diabetes. Family and friends were the source of information for diabetes and its associated symptoms, which mentally prepared the participants for healthcare facility visits and arriving at their diabetes diagnosis.

After being diagnosed with diabetes, many of the participants in our study believed that diabetes was a curable disease. A realm of myths about the cause of diabetes was cited by study participants. Some attributed cause of their diabetes to herbal medicine use, depression, and as a consequence of medication side effects.

Self-perception about their health is a strong facilitator that emerged from this study. In the case of chronic diseases, illness perceptions can shape a positive framework that promotes self-care (47). Fear of complications due to poorly controlled diabetes motivates study participants to adhere to their therapeutic regimens. Broadbent et al. (48) also report similar findings, in which adherence to medication, physical activity, and diet is significantly influenced by patients' diabetes perceptions.

Several barriers to self-care practices emerge from this study. These barriers include financial constraints, the hectic nature of their job, physical limitations, needle phobia, a food-loving nature, and extreme weather conditions. Unintentional non-adherence to medicine due to financial constraints and being over-occupied with a job among Pakistani people suffering from chronic diseases are also reported in another recently published study (30). Injection site pain, unaffordability, and being fed up with routine medicine intake significantly reduces medication compliance and frequency of blood glucose testing.

Cravings for specific foods coupled with unaffordability are among serious challenges that make dietary adjustments difficult for people with diabetes. High costs associated with healthy food choices make it difficult to adopt recommended dietary practices for patients with chronic diseases with a low socioeconomic status (27, 29).

Physical activity in people with diabetes is an important aspect of effective glycemic control and controlling the progression of the disease. Adopting healthy lifestyle modifications, especially physical activity, are frequently reported barriers in many studies (49, 50). Similar to the finding of Lawton et al. (28), in our study, the hectic nature of the job, suffering from comorbid conditions, and extreme weather conditions are commonly reported barriers to physical activity. Several myths are also observed, such as respondents (especially housewives) thinking that their household work was a fair replacement for their exercise.

Our study findings have some practical implications for the healthcare system of Pakistan. First, the inadequate knowledge about a healthy diet and the importance of exercise necessitates healthcare providers to educate their patients about these important aspects of diabetes self care by dedicated face-to-face educational sessions supplemented with informational leaflets and other relevant materials. Second, self-care education must also include information about the causes, complications, and prognosis of diabetes and should be tailored to the cultural perspective and individual patient needs.

### Limitations

Although our study presents new insights into the practices and experiences of T2DM patients in urban areas of Pakistan, there are a few limitations. First, being a qualitative study, one of the limitations is its possible selection bias. Second, there is gender asymmetry in our study participants. Third, self-care practices have not been explored with respect to socioeconomic status and educational background of the study participants. We planned to recruit an equal number of male and female T2DM patients, but due to a higher proportion of female patients at the data-collection sites, more females volunteered for the study. However, it is important to bear in mind the qualitative design of the study, in which the objective of the study is in-depth exploration of problem rather than generalizability.

### Conclusion

Overall, study participants demonstrated poor knowledge about diet planning, the importance of regular exercise, blood sugar testing, and hypoglycemia management. The interviewees also demonstrated the need for counseling by their healthcare providers for diabetes-related self-care practices. Barriers to self care received more prominence in comparison to the facilitating factors. Thus, catering to the informational needs of people with diabetes by an individualized and culturally sensitive self-care educational program should be considered an ideal approach to achieve the desired therapeutic outcomes. Patient education and motivation for appropriate diabetes self care are of paramount importance to improve patients' disease knowledge and self-care practices. The findings of this study will help in designing culturally appropriate and patient-tailored self-care educational interventions for people with diabetes in Pakistan.

## Data Availability Statement

The datasets used and analyzed during the current study will be made available by the corresponding author upon reasonable request.

## Ethics Statement

The studies involving human participants were reviewed and approved by the Monash University Human Research Ethics Committee (MUHREC; Approval Number 7767) and the data collection centers in Pakistan. The patients/participants provided their written informed consent to participate in this study.

## Author Contributions

AB and TK: study concept and design. AB: data collection. AB, TK, and EZ: analysis or interpretation of data. AB, TK, and EZ: thematic analysis. AB, TK, B-HG, CL, K-GC, and SZ: administrative and technical or material support. TK, B-HG, SZ, K-GC, and CL: study supervision. AB: drafting of manuscript. TK, B-HG, SZ, K-GC, and CL: critical revision of the manuscript for important intellectual content.

## Conflict of Interest

The authors declare that the research was conducted in the absence of any commercial or financial relationships that could be construed as a potential conflict of interest.
