# Supplementary material for: Type 2 Diabetes Patients' Perspectives, Experiences, and Barriers Toward Diabetes-Related Self-Care: A Qualitative Study From Pakistan
Source: Front Endocrinol (Lausanne). 2020 Nov 27;11:534873. doi: 10.3389/fendo.2020.534873 (PMC7729167; doi:10.3389/fendo.2020.534873)
Supplement: Supplementary file 1 [file Data_Sheet_1.PDF]

INTERVIEW GUIDE

Introductory question

1. Let's talk about your diabetes, how you were diagnosed with diabetes and where do you go to get the information about your diabetes?
2. How do you take care of your diabetes?

Practices towards diabetes medication

1. When do you take your medicine?
   Probe:
   • Do you understand how to follow your medication?
2. How do you identify which of your medications are used to treat diabetes?
3. Do you ever decide not to take care of your diabetes?
   Probe (Intentional / unintentional):
   - Do you worry about side effects?
   - No reason to continue adhering to treatment plan due to fatalism
   - Regular exercise and strict dietary precautions replaces medication requirement
   - Busy
   - Forgetful
   - Lack of skills
   - No attempts were made to rectify the behavior
   - Tired of taking medicines
   - Feeling deprived
4. What would help you stick to your treatment plan?

Knowledge and practices towards self-monitoring of blood glucose

1. What blood sugar level has your doctor suggested is good for you?
2. How you get your blood glucose level checked? (Home, clinic, frequency, glucose monitor)
3. Do you face any problems when checking your blood sugar?
4. What do you do when you feel shaky, hungry and sweaty or when you feel thirsty, tired and weak or what do you do when you don't feel well?

Knowledge and practices towards diet management

1. What do you think about controlling your blood sugar through healthy food choices?
   Probe:
   - What do you mean? In what way will ______________ make it easier for you?
2. What food choices would make a difference in your blood sugar control?
3. Current knowledge about diet plan was obtained from physician or a dietitian recommended by him?
4. Do you feel difficulty in eating less but more frequently?
5. What makes you feel difficult in adopting diet plan?
   Probe:
   - Favorite foods
   - Job nature
   - Family get together / social traditions
   - affordability

Knowledge about exercise and barriers to it

1. What sort of physical activity recommended to you by your physician? Are you following it?

Probe:

- Regularity
- Confusing their job exertion as replacement for regular exercise
- influence of weather

Continuity of care

1. For the following questions, I want you to think about those visits to your doctor related to your diabetes. If you saw a doctor for both your diabetes and another condition, that would still count as a visit related to your diabetes. What makes you decide when you should go to visit your doctor for follow-up care?

Probe:

- What types of health problems make you visit the primary care doctor?
- How often do you visit your primary care doctor for regular checkups?
- How often do you visit other health care providers because your primary care doctor has referred you?

2. How often does your primary care doctor ask about other medications you are taking?
